# Structure and surgical dissection layers of the bare area of the liver

**DOI:** 10.1186/s12893-020-00830-8

**Published:** 2020-07-31

**Authors:** Takamichi Ishii, Satoru Seo, Takashi Ito, Satoshi Ogiso, Ken Fukumitsu, Kojiro Taura, Toshimi Kaido, Shinji Uemoto

**Affiliations:** Division of Hepato-Biliary-Pancreatic Surgery and Transplantation, Department of Surgery, Graduate School of Medicine, Kyoto University, 54 Kawahara-cho Shogoin, Sakyo-ku, Kyoto, 606-8507 Japan

**Keywords:** Bare area, Liver, Diaphragm, Cadaver, Laennec’s capsule

## Abstract

**Background:**

The bare area was reportedly formed by direct adhesion between the liver and diaphragm, meaning that the bare area lacked serosal components. This study aimed to analyze the structure of the bare area by an integrated study of surgical and laparoscopic images and pathological studies and describe surgical procedures focusing on the multilayered structure.

**Methods:**

Several surgical specimens of hepatectomy were analyzed histologically to evaluate the macroscopic structure of the bare area. Laparoscopic images and cadaver anatomy of the bare area were also examined.

**Results:**

The multilayered structure of the bare area comprised the liver, sub-serosal connective tissue, liver serosa, parietal peritoneum, retroperitoneal connective tissue, epimysium of the diaphragm, and diaphragm, in order from the liver to the diaphragm. The liver serosa and the parietal peritoneum fused with each other. This multilayered structure of the bare area is observed almost constantly. There are two layers in the dissection of the bare area in surgical procedures, an outer layer of the fused peritoneum (near the diaphragm) and an inner layer of the fused peritoneum (near the liver). Laparoscopic images enabled us to recognize the multilayered structure of the bare area.

**Conclusions:**

Histopathological findings showed the bare area to be a multilayered structure. In cases where tumors are located underneath the bare area, it could be important to dissect the bare area, with careful attention to its multilayered structure. Surgical dissection of the bare area in the outer layer of the fused peritoneum could allow a sufficient safety margin.

## Background

In the process of human development, a liver grows, rotates, and adheres to the right diaphragm [1, 2]. The attachment site of the liver to the right diaphragm is known as the bare area. The liver surface under the bare area is thought to lack a peritoneal covering in adults [3, 4]. The term, bare area, originates from this recognition. However, the bare area has a multilayered structure, namely a fused peritoneum generated from the hepatic visceral peritoneum and the diaphragm parietal peritoneum. In hepatic surgery, the bare area sometimes needs to be dissected to provide mobilization of the right liver. Especially when the tumors are located below the bare area, it could be important to recognize the multilayer structure when surgically approaching the tumors, which might result in the prevention of tumor cell dissemination.

The aim of this paper was to analyze the structure of the bare area by an integrated study of laparoscopic surgical images and histopathological studies and to describe surgical procedures. We focused on the relationship between the liver and diaphragm in the bare area, showing the multilayered structure of the bare area, because this part of the bare area is important in the mobilization of the right liver.

## Methods

### Representative patients

#### Case 1

**Fig. 1:**
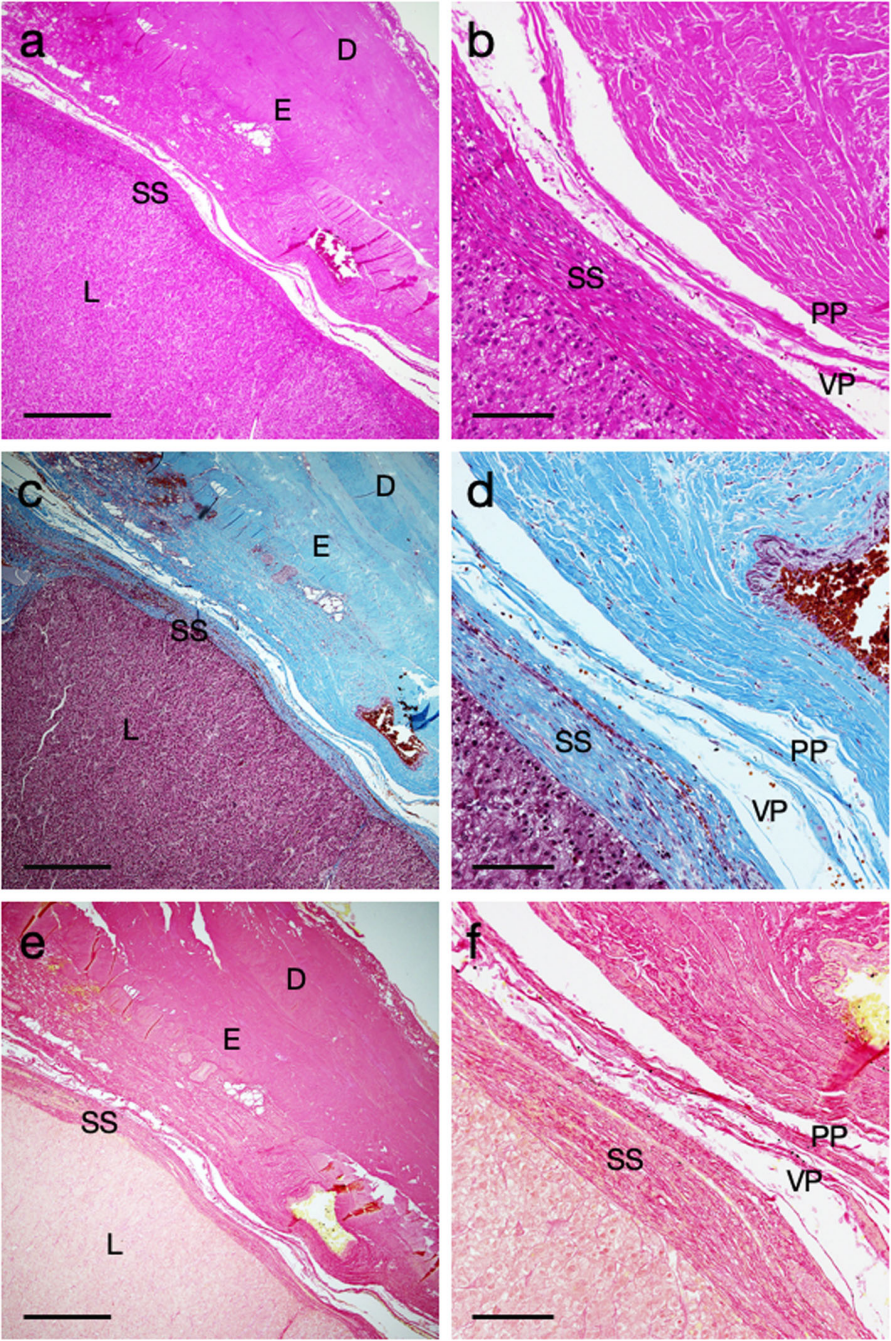
Histopathological images of the bare area including the liver and the diaphragm (Case 1). **a** and **b** Hematoxylin-eosin staining. **c** and **d** Masson trichrome staining. **e** and **f** Sirius red staining. L; liver, SS; sub-serosal connective tissue, VP; visceral peritoneum, PP; parietal peritoneum, E; epimysium, D; diaphragm. Bars in (**a**, **c**, and **e**) indicate 500 μm, and bars in (**b**, **d**, and **f**) indicate 100 μm

A 75-year-old man underwent a right hemi-hepatectomy for a huge hepatocellular carcinoma with partial resection of the right diaphragm. The right liver was removed without dissection of the bare area. The histological findings are shown in Fig. 1.

#### Case 2

**Fig. 2:**
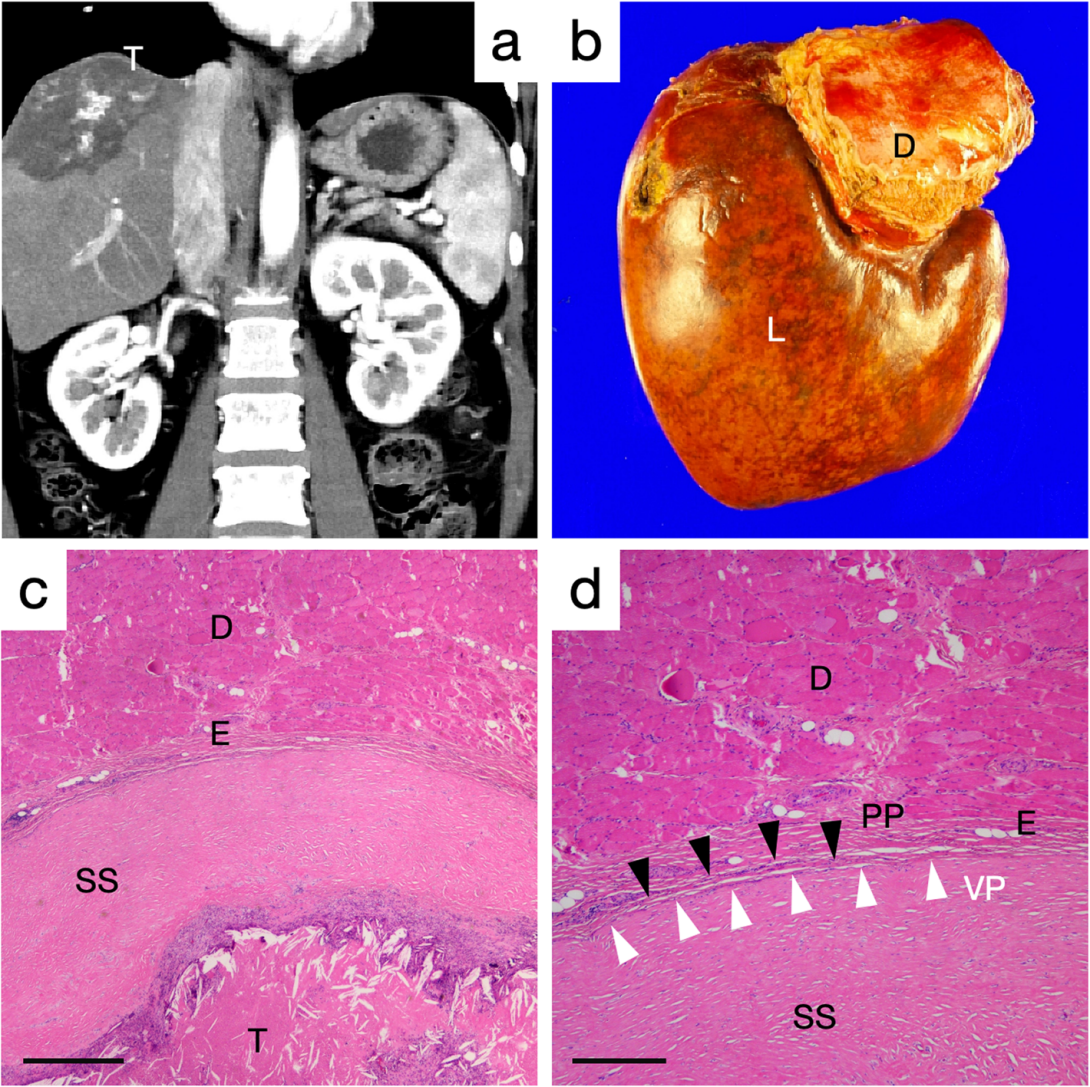
The preoperative computed tomography, resected specimen, and histological findings of the 45-year-old woman with a huge colorectal cancer liver metastasis (Case 2). **a** Preoperative computed tomography image. **b** Macroscopic finding of the resected liver with the diaphragm. **c** and **d** Hematoxylin & eosin staining. T; tumor, L; liver, SS; sub-serosal connective tissue, VP; visceral peritoneum (white arrowheads), PP; parietal peritoneum (black arrowheads), E; epimysium, D; diaphragm. A bar in c indicates 500 μm, and a bar in d indicates 100 μm

A 45-year-old woman had a synchronous huge metastatic liver tumor from colon cancer just underneath the right diaphragm. She underwent laparoscopic right hemicolectomy, following chemotherapy for 5 months. Subsequently, she underwent right hemi-hepatectomy with partial resection of the right diaphragm. The preoperative computed tomography (CT), resected specimen, and histological findings are shown in Fig. 2.

#### Case 3

A 64-year-old woman underwent laparoscopic right hemi-colectomy for colon cancer. Five months after the operation, a metastatic liver tumor, 15 mm in diameter, was found at hepatic segment 7. She underwent chemotherapy for 6 months and then underwent laparoscopic partial hepatectomy. The preoperative CT, intraoperative findings, and histological findings are shown in Fig. 5 and Fig. 6.

Written informed consent was obtained from all patients in this study in accordance with the ethical guidelines of Kyoto University Hospital. This study was approved by the ethics committee of Kyoto University (approval number: R-1721) and performed in accordance with the 1964 Helsinki declaration and its later amendments.

### Surgical procedure and patient care

All patients underwent multi-detector CT scan before the operation. Three-dimensional images were reconstructed from the CT data using SYNAPSE VINCENT (Fujifilm, Tokyo, Japan).

The surgical procedures used in the study have been described in previously published articles [5–7]. In open hepatectomy, hepatic resection was performed using a Mercedes incision or an inverted L-shaped incision. The liver was mobilized, and an intraoperative ultrasound was performed to assess the lesions. An intermittent Pringle maneuver, or selective vascular clamping if necessary, was applied to occlude the blood inflow of the liver. Hepatic parenchymal resection was performed using CUSA Excel (Integra LifeSciences, Plainsboro, NJ, USA) and bipolar cautery equipped with a channel for water dripping.

In laparoscopic hepatectomy, the patient was placed in the left half-lateral decubitus position to expose the lateral and posterior aspect of the right lobe. Pneumoperitoneal pressure by carbon dioxide was maintained at 8–12 mmHg. For the assessment of the tumor location and its relationship with the vasculature, intraoperative contrast-enhanced ultrasonography was routinely performed. The transection of the liver surface was performed using an ultrasonically activated scalpel, and the parenchymal resection was performed by clamp crush methods using an intermittent Pringle maneuver. Bipolar electrocoagulation was performed for minor bleeding.

### Cadaver anatomy

The study utilizing donated cadavers was approved by the ethics committee of Kyoto University (approval number: R-1785). One liver was studied in a Thiel-embalmed female cadaver in her seventies. The bare area of the liver was dissected sharply from the diaphragm using surgical scissors, and the right liver surface was exposed, changing the dissection layers.

### Histological examination

Surgical materials were fixed by formalin for approximately 48 h. After thorough rinsing, the tissues were then embedded in paraffin and sectioned into 3 μm-thick sections. Hematoxylin-eosin (HE) staining, Masson trichrome staining, and Sirius red staining were performed according to the standard protocol [8–10].

## Results

### Histological findings of the bare area of the liver

In case 1 patient, the histological specimens were obtained from the bare area. H&E staining revealed no direct tumor invasion into the diaphragm. The serial sections were also stained by the Masson trichrome and Sirius red stains to clarify the structure of the connective tissues between the liver and the diaphragm. Sparse connective tissues were lying in a width of 100–200 μm between the liver and the diaphragm in low-magnification micrographs (Fig. 1a, c, and e). The high-magnification micrographs showed that the liver parenchyma was covered with liver serosa (visceral peritoneum) and approximately 100 μm-thick dense sub-serosal tissues. In addition, the diaphragm tendon and its epimysium were covered with parietal peritoneum (Fig. 1b, d, and f). In case 2 patient, a huge liver tumor was located underneath the right diaphragm (Fig. 2a). The right liver was resected with the partial right diaphragm, which had adhered to the liver tightly (Fig. 2b). H&E staining revealed no direct tumor invasion into the diaphragm. The necrotic tumor was located just below the dense sub-serosal tissues of the liver (Fig. 2c). The diaphragm and liver were covered with a monolayer of parietal peritoneum and visceral peritoneum, respectively (Fig. 2d). Unlike the case 1 patient, the sub-serosal tissues of the liver were hypertrophic. This was done partially because of the necrotic tumor after chemotherapy.

**Fig. 3:**
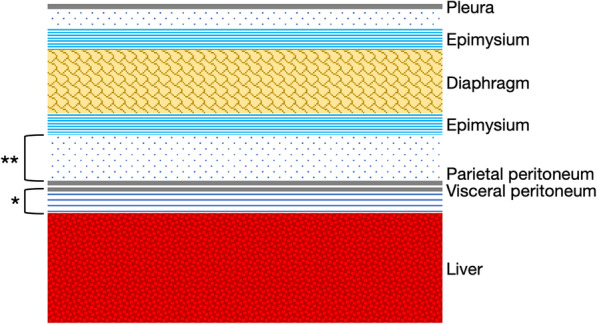
Schema of the multilayered structure of the bare area. The visceral peritoneum fuses with the parietal peritoneum. *; inner layer of the fused peritoneum, **; outer layer of the fused peritoneum.

The multilayered structure of the bare area was composed of the liver parenchyma, sub-serosal connective tissue, liver serosa, parietal peritoneum, retroperitoneal connective tissue, epimysium of the diaphragm, and the diaphragm, in order from the liver to the diaphragm (Fig. 3). There were 200–300-μm-thick connective tissues between the liver parenchyma and epimysium of the diaphragm of the bare area, and the liver serosa was close to the parietal peritoneum that caused peritoneal fusion. There are two layers in the dissection of the bare area in surgical procedures, an outer layer of the fused peritoneum (near the diaphragm) and an inner layer of the fused peritoneum (near the liver).

### Macroscopic findings of the bare area of the liver

**Fig. 4:**
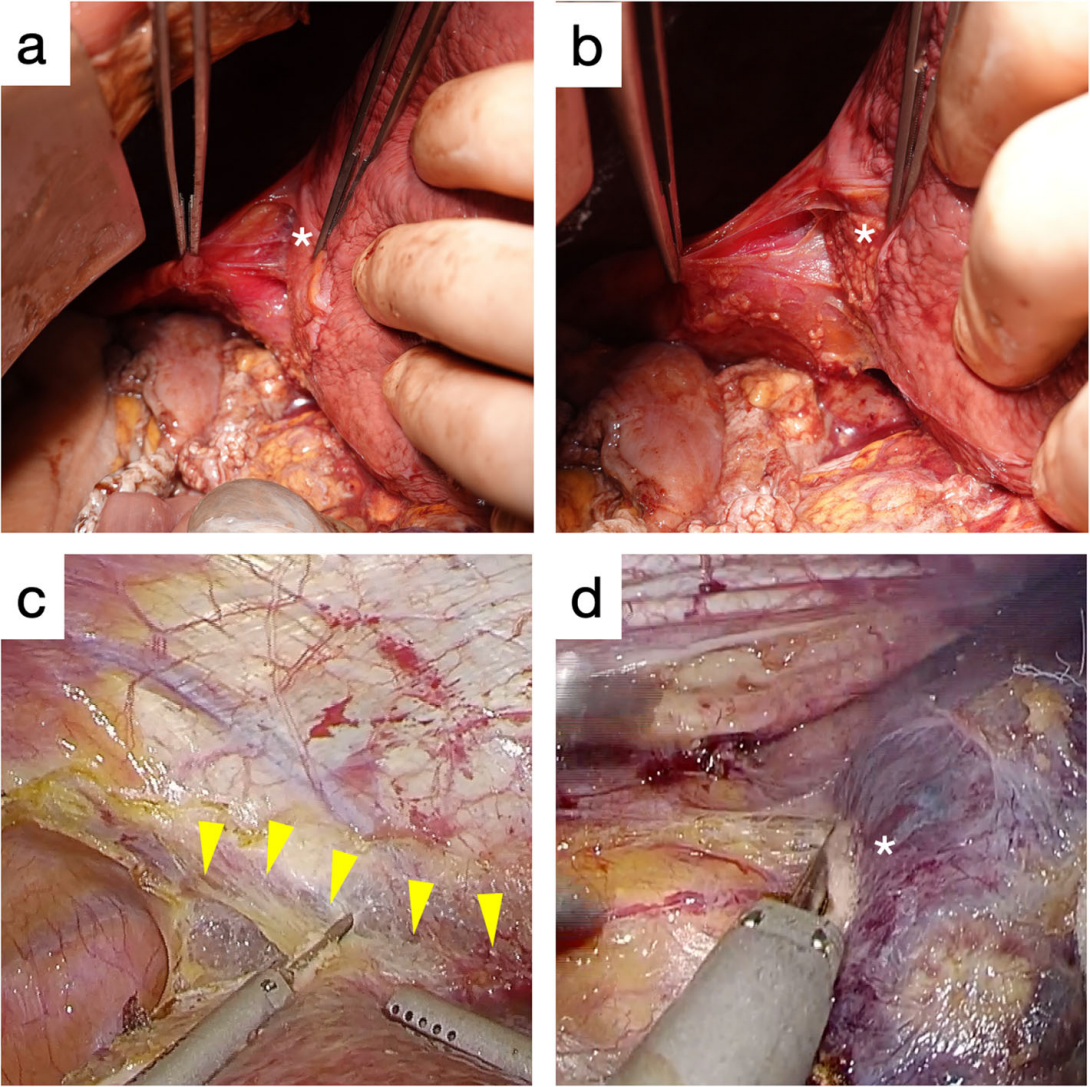
**a** The liver surface (*) is covered with glossy white connective tissues when dissected in the outer layer of the fused peritoneum (cadaver anatomy). **b** When dissected in the inner layer of the fused peritoneum, the liver surface (*) is naked compared to the adjacent surface with the liver serosa (cadaver anatomy). **c** Intraoperative image of a laparoscopic partial hepatectomy for hepatocellular carcinoma in a 69-year-old man: The line between the outer layer and the inner layer of the fused peritoneum is shown by yellow arrowheads. **d** Intraoperative image of a laparoscopic partial hepatectomy for colorectal liver metastasis in a 71-year-old man: When the inner layer of the fused peritoneum is dissected, the liver surface (*) covered only by thin connective tissue is exposed

In order to examine the possibility of recognizing the multilayered structure of the bare area intraoperatively, a cadaver and two cases of laparoscopic hepatectomy were analyzed. In the cadaver study, when dissecting the bare area near the diaphragm, the surface of the liver was seen to be covered with dense fibrous tissues (Fig. 4a). On the other hand, when dissecting near the liver, the raw surface of the liver was exposed (Fig. 4b). In the case of laparoscopic bare area dissection, surgeons could sometimes recognize the line between the spare connective tissue on the diaphragm side, and the dense fibrous tissue on the liver side when the liver exerted traction is in the counter direction of the diaphragm (Fig. 4c). When dissecting the bare area near the liver (the inner side of the fused peritoneum), the liver surface covered only by a thin connective tissue was exposed (Fig. 4d). Therefore, it was possible to recognize the multilayered structure of the bare area and select the dissection layer under the magnified view of laparoscopy.

### Case presentation of a colorectal liver metastatic tumor located directly beneath the right diaphragm

**Fig. 5:**
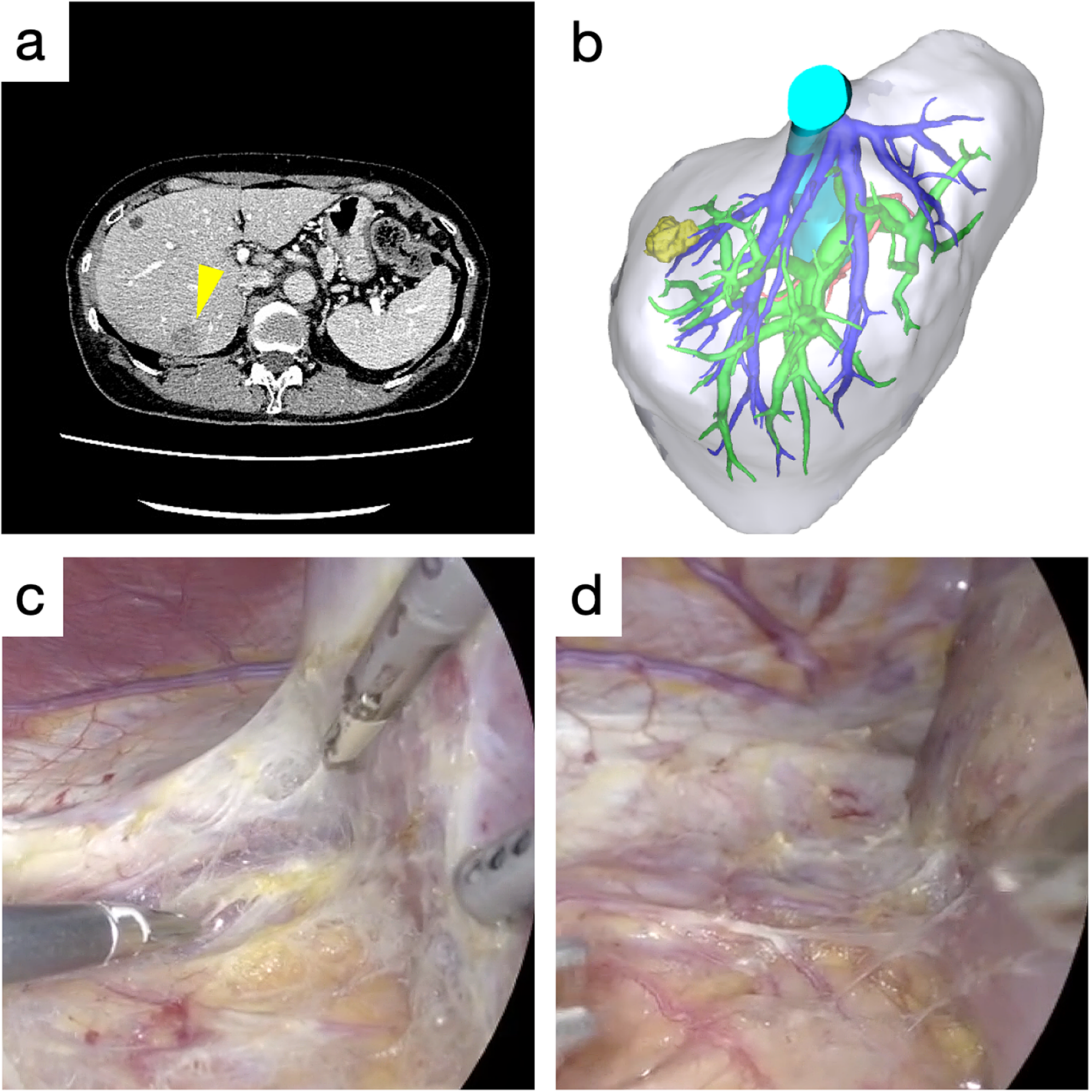
Preoperative and intraoperative images of the 64-year old woman with a colorectal cancer liver metastasis (Case 3). **a** Preoperative computed tomography image. The tumor is indicated by a yellow arrowhead. **b** Three-dimensional image reconstructed by SYNAPSE VINCENT. The tumor is indicated in yellow. **c** Laparoscopic partial hepatectomy was performed. The bare area dissected is the outer layer of the fused peritoneum. **d** The right liver upon dissection revealed a dense white connective tissue

The case 3 patient is presented to demonstrate the importance of selecting the appropriate dissection layer of the bare area for good surgical therapeutic effect. Preoperative CT revealed a tumor 10 mm in diameter, located directly underneath the right doom of the diaphragm (Fig. 5a and b). Because of a possible direct invasion of the tumor into the right diaphragm, the bare area was dissected carefully near the diaphragm (the outer layer of the fused peritoneum) (Fig. 5c). There was no gross invasion observed, and the right liver was dissected from the diaphragm at the outer layer of the fused peritoneum with a dense white connective tissue (Fig. 5d). The multilayered structure of the bare area was recognizable with the high definition images of laparoscopy.

**Fig. 6:**
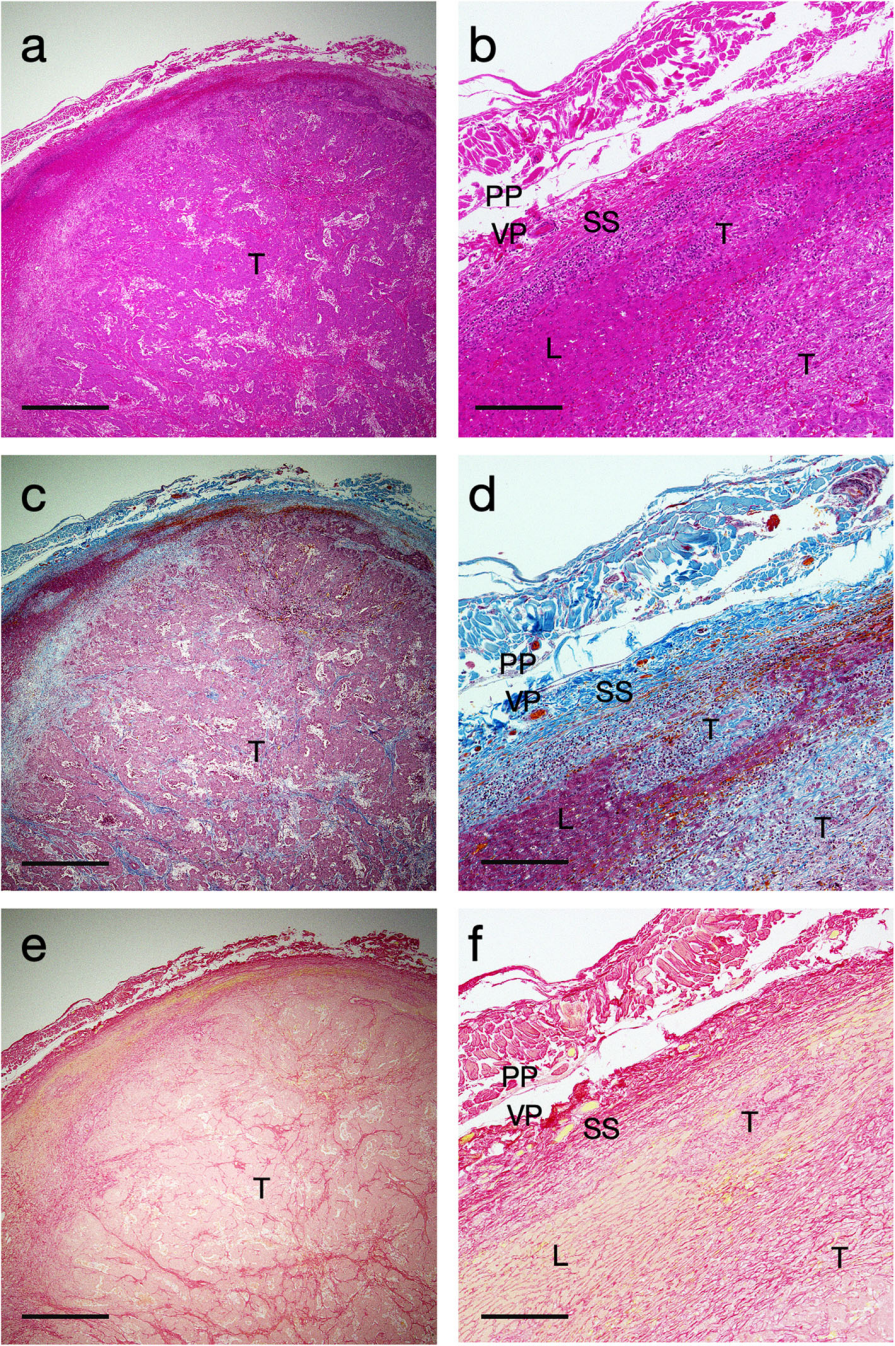
Histopathological images of the liver surface of the resected specimen. **a** and **b** Hematoxylin-eosin staining. **c** and **d** Masson trichrome staining. **e** and **f** Sirius red staining. T; tumor, L; liver, SS; sub-serosal connective tissue, VP; visceral peritoneum, PP; parietal peritoneum. Bars in (**a**, **c**, and **e**) indicate 1000 μm, and bars in (**b**, **d**, and **f**) indicate 200 μm

The low-magnification micrographs revealed that the tumor was located quite close to the liver serosa, although there was no direct invasion into the diaphragm (Fig. 6a, c, and e). However, tumor cells expanded to the sub-serosal connective tissue in the high-magnification micrographs (Fig. 6b, d, and f). In this surgery, the bare area was dissected at the outer layer of the fused peritoneum, and the parietal peritoneum and the partial retroperitoneal connective tissue were removed with the liver. Therefore, surgical margins of approximately 500 μm were reserved.

## Discussion

The present study results revealed that the bare area has a multilayered structure. The bare area has been recognized as a hepatic surface area lacking the peritoneum [3, 4]. The term “bare area” is derived from this recognition. However, in the fetal hepatic developmental process, the liver enlarges, rotates, and adheres to the diaphragm, resulting in the formation of the bare area [1, 2]. This indicates that even the liver surface of the bare area has the peritoneum [11]. The histopathological examination in this study revealed that the bare area had a multilayered structure, which was observed almost constantly. The parietal peritoneum covering the diaphragm and the visceral peritoneum, which is the liver serosa, is in close proximity and fused. There is a relatively dense connective tissue on the liver side and sparse connective tissue on the diaphragm side of the fused peritoneal peritoneum. The structure composed of dense connective tissue on the liver side and liver serosa is the Laennec’s capsule [12, 13]. Because the liver surface of the bare area is covered with the Laennec’s capsule, as shown in our study, the phrase “bare area” may not be appropriate.

### Application of the histological findings in surgical procedures

Recognition of the multilayer structure of the bare area during surgery: Cadaver anatomy revealed a glossy white connective tissue attached to the liver surface when the diaphragm side was dissected. On the other hand, the dissection on the liver side exposed to the raw liver surface. Therefore, it is possible to dissect the bare area by changing the dissection layer. It might be difficult to recognize the fusion peritoneum itself because both the parietal peritoneum and the liver serosa are a monolayer peritoneum structure. However, under laparoscopic magnification, the borderline between the loose connective tissue of the outer layer of the fused peritoneum and the dense connective tissue of the inner layer can be recognized. Especially in patients with liver cirrhosis, this boundary might be easy to recognize because the Laennec’s capsule has often thickened in such patients. Therefore, it is possible to select the dissection layer at least inside and outside the fusion peritoneum.

Selection of dissection layers of the bare area’s multilayers could affect surgical control of tumors: When the tumor is located just below the diaphragm dome, as shown in the representative case presented in this study, the tumor cells could invade very close to the liver surface. Consciously dissecting the outer layer of the fused peritoneum could secure a safe surgical margin from tumors and possibly result in the prevention of the dissemination. If the tumor is located deep inside the liver or the surgery is not for a malignant tumor, it may be better to dissect the bare area along with the inner layer of the fused peritoneum. This is because the diaphragm is so thin that it may be injured during the surgical procedure. Diaphragmatic injuries could make surgical procedures more difficult, especially in laparoscopic surgery.

The bare area is surrounded by the right coronary ligament, right triangular ligament, hepatorenal ligament, and inferior vena cava (IVC) [3, 4]. In this study, the peritoneal relationship between the liver and IVC remains to be elucidated. Further studies using cadavers will be needed to clarify the peritoneal relationship between the liver and IVC.

## Conclusion

The bare area is a multilayered structure. There are dissection layers on the outside and inside of the fused peritoneum, respectively. These dissection layers are visible macroscopically. If tumors are located in close proximity to the diaphragm, dissection of the bare area should be performed with attention to this multilayered structure.

## Data Availability

The datasets generated and analyzed during the current study are available from the corresponding author on reasonable request.

## Abbreviations

CT: Computed tomography
HE: Hematoxylin-eosin
IVC: Inferior vena cava
